# Supplementary material for: Evaluation of transplacental transfer of mRNA vaccine products and functional antibodies during pregnancy and early infancy
Source: Res Sq. 2021 Dec 15:rs.3.rs-1150427. Preprint. [Version 1] doi: 10.21203/rs.3.rs-1150427/v1 (PMC8687466; doi:10.21203/rs.3.rs-1150427/v1)
Supplement: Supplement 1 [file ed822670c7572592a0df3e9d.pdf]

Supplementary Material

Table of Contents

	Page
Table S1	2
Table S2	2
Table S3	3
Figure S1	4

Table S1: Maternal Demographics

Study ID	Gestational Age at Delivery	Infant Sex	Vaccine Type	Gestational Age at Dose 1	Maternal Age
11012	37.71	Female	Moderna	26.572	36
11018	37.43	Female	Moderna	36	30
11026	37.57	Female	Moderna	31.858	33
11033	20.43	Male	Pfizer	13.143	29
11036	38.71	Male	Moderna	31	40
11038	39.86	Female	Moderna	31.286	35
11040	39.29	Male	Moderna	33.715	35
11045	38.86	Female	Pfizer	30.572	36
11047	39.29	Male	Moderna	31.572	36
11048	39.14	Male	Pfizer	25.286	33
11058	40.14	Female	Moderna	33.858	34
11059	40.14	Male	Moderna	30.429	36
11062	40.29	Male	Moderna	30.715	36
11063	37.57	Female	Moderna	25	29
11085	41.14	Male	Pfizer	40.286	34
11091	39.57	Female	Pfizer	28.143	36
11123	39.14	Male	Pfizer	35	38
11126	39.14	Female	Pfizer	36	39
11128	39.71	Male	Pfizer	37	33
11137	40.57	Male	Moderna	37	33

Table S2: Serial dilution of vaccine cDNA

pg Vaccine cDNA	Moderna Ct	Pfizer Ct
10000.00	6.578	6.696
3333.33	8.247	12.266
1111.11	11.403	12.804
370.37	12.799	15.355
123.46	14.327	16.314
41.15	16.217	19.397
13.72	18.430	19.727
4.57	20.503	21.579
1.52	23.315	24.646
0.51	23.337	23.998
0.17	24.259	26.110
0.06	25.510	27.840

*Sensitivity to 1.5 pg/uL

Table S3: Spike protein western blot and vaccine mRNA PCR results

	Spike Protein Detection (Western blot)			Spike mRNA Detection (qRT-PCR)		
Participant	Placenta	Maternal Blood	Cord Blood	Placenta	Maternal Blood	Cord Blood
11012	Negative	Negative	Negative	Negative	Negative	Negative
11018	Negative	Negative	Negative	Negative	Negative	Negative
11026	Negative	Negative	Negative	Negative	Negative	Negative
11033	Negative	Negative	Negative	Negative	Negative	Negative
11036	Negative	Negative	Negative	Negative	Negative	Negative
11038	Negative	Negative	Negative	Negative	Negative	Negative
11040	-	Negative	Negative	-	Negative	Negative
11045	Negative	Negative	Negative	Negative	Negative	Negative
11047	-	Negative	Negative	-	Negative	Negative
11048	Negative	Negative	Negative	Negative	Negative	Negative
11058	Negative	Negative	Negative	Negative	Negative	Negative
11059	-	Negative	Negative	-	Negative	Negative
11062	Negative	Negative	-	Negative	Negative	-
11063	Negative	Negative	Negative	Negative	Negative	Negative
11085	-	-	-	-	-	-
11091	Negative	Negative	Negative	Negative	Negative	Negative
11123	Negative	Negative	Negative	Negative	Negative	Negative
11126	Negative	Negative	Negative	Negative	Negative	Negative
11128	Negative	Negative	Negative	Negative	Negative	Negative
11137	Negative	Negative	-	Negative	Negative	Negative

- Missing sample

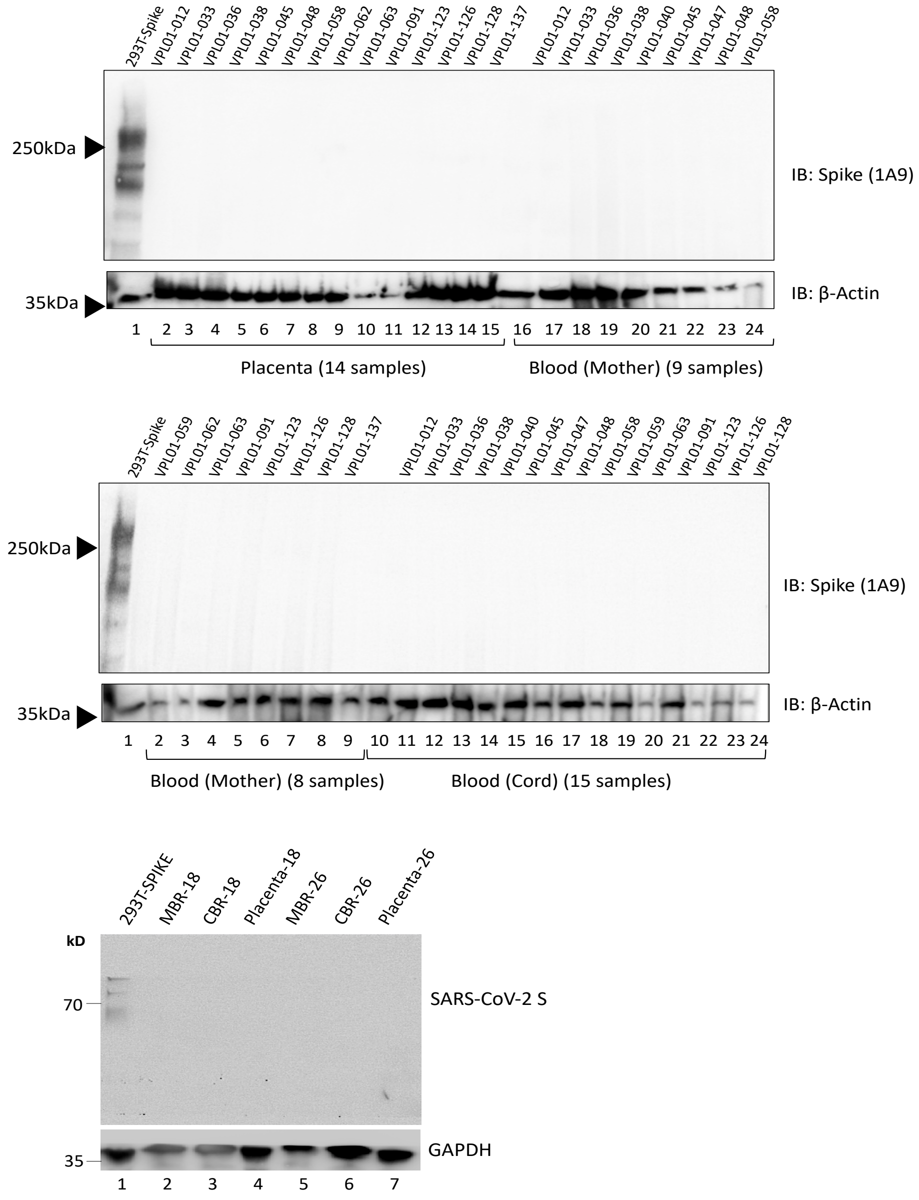

Supplementary Figure 1: Spike protein Western blot

Western blot of maternal blood at delivery, placenta tissue, and cord blood compared to Spike protein positive control
